# Measuring transnational social fields through binational link-tracing sampling

**DOI:** 10.1371/journal.pone.0253042

**Published:** 2021-06-14

**Authors:** Marian-Gabriel Hâncean, Miranda Jessica Lubbers, José Luis Molina

**Affiliations:** 1 Department of Sociology, University of Bucharest, Bucharest, Romania; 2 Department of Social and Cultural Anthtropology, Universitat Autònoma de Barcelona, Barcelona, Spain; Centre National de la Recherche Scientifique, FRANCE

## Abstract

We advance bi-national link-tracing sampling design, an innovative data collection methodology for sampling from so-called “transnational social fields”, i.e. transnational networks embedding migrants, returned migrants and non-migrants. This paper describes our contributions to this methodology and its empirical implementation, and evaluates the features of the resulting networks (sample), with the aim to guide future research. We performed 303 face-to-face structured interviews on sociodemographic variables, migration trajectories and personal networks of people living in a Romanian migration sending community (Dâmbovița) and in a migration receiving Spanish town (Castellón). Inter-connecting the personal networks, we built a multi-layered complex network structure embedding 4,855 nominated people, 5,477 directed ties (nominations) and 2,540 edges. Results indicate that the link-tracing nomination patterns are affected by sex and residence homophily. Our research contributes to the emerging efforts of applying social network analysis to the study of international migration.

## Introduction

Migration is not randomly distributed across the globe. Specific *binational migration corridors* can be identified, such as Mexico-US (the largest corridor between 1990–2000 and between 2000–2010) and Syria-Turkey (far the largest between 2010–2017) [1]. Inside such corridors, due to *migration networks* or *chain migration*, we can detect flows from one specific geographical area within a country of origin to a specific area within a destination country [2]. This network mechanism implies that once a small number of people from a specific area have settled in a certain destination area, it is easier for others to undertake the same trajectory. Migrants of former waves can pass information to help them start their migration project, find employment or housing, understand the national legislation and administrative frameworks.

Such regional migration corridors affect not only migrants, but also non-migrants and returnees, through exchanges of information, remittances, services, and “culture”. To investigate these exchanges and their effects, scholars of transnationalism have proposed the term “*transnational social field*” (TSF), defined as “an unbounded terrain of interlocking egocentric networks that extends across the borders of two or more nation-states and that incorporates its participants in the day-to-day activities of social reproduction in these various locations” [3]. TSFs capture “immigrants, persons born in the country of origin who never migrated, and persons born in the country of settlement of many different ethnic backgrounds” [4]. Thus, TSFs are defined on the basis of migrants who move between geographically defined places of origin and destination, and only include return migrants and non-migrants insofar they are connected to the focal actors by a relevant social relationship [5].

TSFs have fuzzy boundaries, both in terms of geography and membership. This is a challenge for constructing samples from such fields. First, migrant populations often lack a *sampling frame*, which means that the size and boundaries of the population (including the geographical dispersion) are unknown to researchers [6]. To this effect, they can be considered *hidden* or *hard-to-reach* populations [7, 8]. Whereas in the case of *known populations* (*i*.*e*., for which a sampling framework exists), traditional probability sampling methods can be applied, these methods lack efficiency in producing reliable samples for *migrant populations*. Additionally, efforts to quantitatively describe migrant populations are severely restricted by a wide range of other challenges, including residential mobility, low availability for home interviewing, reluctance to research participation and to revealing personal data, lack of trust, high sensitivity to specific research topics, official language barriers, cultural differences, legal status, social security affiliation [9]. On top of that, TSFs do not only include migrant populations but also non-migrants and returnees insofar they are connected to the focal migrants. As it is *a priori* unknown to researchers who is and who is not associated with migrants in the specific area of destination, these individuals can only be indirectly sampled, through the referral of others.

Solutions to the impracticability of traditional probability designs to the study of migrant populations are still in an infant stage of development. At the same time, despite the network character exhibited by the migration processes, substantive research into these networked processes have proven to be rare until recently [10]. Consequently, little is known about the structure and composition of these transnational networks, and their effects on, for example, mobility patterns, identity formation, the emergence of migrant entrepreneurship, the transnational exchange of care, and the circulation of remittances. In this context, the present study contributes to the recent efforts of quantitatively describing migrant populations and migration networks [5] by adopting a network sampling design (*i*.*e*., employing a chain-referral data collection strategy), a design that uses social networks to obtain convenience and representative samples from hidden populations. The design intends to sample from a TSF with the aim to increase the understanding of migration processes and patterns. Our research extends already existing methodologies [11–13] in terms of the data collection process (*i*.*e*., implementation of simultaneously instead of sequentially multi-sited data collection) and of providing more thorough description of migrants’ networks (*i*.*e*., elicitation of perceived relationships among a respondent’s network contacts, collection of diverse attribute data as to increase the accuracy and robustness of identifying across nominations the unique individuals within the network). Thus, we not only replicate results (with reference to previous research), but also bring forth new insights on migration within transnational networks. Moreover, so far, descriptions of practical implementation of such methodologies are lacking, despite their obvious relevance for guiding future studies. Therefore, this paper also contributes to the literature by describing the empirical implementation of the methodology in depth.

We studied the TSF created by Romanian immigrants from Dâmbovița (a Romanian county with a resident population of 487,115 people, situated at 78 km North-West of Bucharest, Romania) to Castellón (a Spanish province of nearly 577,000 inhabitants, situated on the Mediterranean coast, where 11% is Romanian). Our method is based on network sampling, a sampling strategy that uses social networks to obtain convenience and representative samples from hidden populations. However, so far, descriptions of the practical implementation of such methodologies are lacking, despite their relevance for guiding future studies. The objective of this paper is to describe the implementation of the methodology for the empirical study of TSFs. Additionally, we evaluate the resulting networks to detect potential biases and shortcomings of the methodology and to serve as an example for data exploration in future work. For example, as we describe below, networks tend to be homophilous, *i*.*e*., people tend to relate with others who are similar to them in demographic characteristics such as sex, which in the case of network recruitment can lead to biased sample. Hence, the second objective of the paper is to describe the type of network that the binational ink-tracing design reveals. Put it differently, what are *the properties* of the migration network visualized with the binational link-tracing design?

In sum, the overall aim of the paper is to present the empirical implementation of the bi-national link-tracing sampling methodology and to evaluate the features displayed by the resulting networks.

The structure of the paper is as follows. Firstly, we review the literature on network-oriented sampling methods for hidden populations. Secondly, we briefly introduce network approaches to measure TSFs. Afterwards, we describe our research design (a binational link-tracing variant) implemented for measuring the TSF wherein Romanian migrants in or returned from Spain (Castellón) as well as their social contacts (relatives, friends and acquaintances) are embedded. We then present the results obtained after sampling from the hidden population of Romanian migrants (the major demographic characteristics of the study participants, the structural and compositional features of the measured TSF). Finally, the paper discusses the implications of our study, some limitations, and future directions.

### Sampling from transnational social fields

#### Network-oriented sampling methods

Migrants and non-migrants in TSFs are typically considered a hidden or hard-to-reach population, *i*.*e*., a population for which the degree of access for collecting data is low. Due to the impracticability of non-network probability sampling designs to the study of hidden populations, *network-oriented sampling methods* (or *link-tracing sampling* methods) have been deployed, which essentially implement chain-referral strategies for collecting data. Initially, link-tracing sampling designs, such as snowball methods [14], were used to construct networks and study social structures [6–8, 15, 16]. Network-oriented sampling designs were rapidly transferred to the study of hidden populations due to their capacity of locating affiliated members [7]. Specifically, as [8] argues, network-oriented sampling methods use a link-tracing or chain-referral strategy of collecting data (*i*.*e*., *chain data*) and allow for eliciting members in hidden populations (such as population of migrants). Researchers’ appeal to this specific class of sampling methods could be explained by the effectiveness of *locating members* of hidden populations, as well as by the superiority in rapidly *increasing* the number of members of a target population in a sample.

The most popular non-probability form of link-tracing method is *snowball sampling* [17]. This method was described by [14] as implying *s* stages and *k* names. Precisely, a small, randomly selected set of individuals from a given population is used as the first phase of the sampling procedure (the *seeds*). Next, each individual in the set is asked to name *k* individuals in the population who are not in the randomly selected set. The *k* people form the second phase and are asked to further name *k* individuals. The *k* people who are not in the first and second phase are then asked to name *k* different individuals. The procedure continues until *s* stages or a specific sample size are achieved [17].

The term snowball sampling is currently used for any method that starts with a small number of (usually not randomly selected) seeds, and asks them for referrals (as many as they can give) until the desired sample size is obtained or saturation is reached. Thus, the number of names per interviewee, the number of stages, and the precise referral chains are not controlled. In effect, despite providing a higher degree of coverage for the cases of hard-to-reach populations (compared to traditional probability sampling methods), snowball designs typically produce convenience samples. Contrary to initial claims that snowball sampling can be used to make statistical inferences [14], multiple sources of biases were shown [16–18]. Precisely, firstly, it was argued that the *initial sample is unlikely to be representative*. Among others, the number of seeds is often too small, and their participation often involved *volunteering*. Second, chain-referral samples were suggested to be *biased toward more cooperative participants*. Thirdly, it was suggested that the attributes of seeds impacted upon additional participants through *homophily*, which is especially troubling when initial subjects are not randomly selected. Fourthly, participants tended to protect their friends by not referring them, particularly when privacy issues are involved, *i*.*e*., a tendency called *masking*. Fifthly, as referrals occur through network ties, individuals with larger personal networks have greater chances of being selected thus being oversampled. Because of these biases, snowball and similar chain-referral samples have been appraised as *convenience samples* (*i*.*e*., non-representative samples).

#### Respondent driven sampling methods

The efforts of transforming link-tracing / chain-referral designs into probability sampling methods are manifest in the work on *respondent driven sampling* (RDS). RDS, invented by Heckathorn, illustrates a class of methods aiming to convert chain-referral sampling into a method of good estimability, by reducing some of its critical biases [6, 7, 16, 17]. RDS is based on Markov chains as well as on a dual system of incentives to drag behavioral compliance on the part of subjects from the target population. According to Heckathorn, firstly, by implementing a Markov modeling peer recruitment process (*memoryless recruitment*), as sample increases one wave after another, an equilibrium sample composition is rapidly achieved. That means the biases of the sample caused by the seeds’ characteristics are eliminated after approximately four waves. Secondly, RDS typically employs a dual incentive system: rewards for being interviewed–*primary incentives*–, as well as for recruiting others–*secondary incentives* (the latter rewards are effective for recruiting less cooperative subjects). Thirdly, an RDS sample is reported to be unbiased when the homophily of each group, represented by different seeds, is equal or when the network size of the participants is controlled. Fourthly, study participants are not required to identify their peers but to recruit them. In effect, *the masking* bias is said to be reduced as respondents are given the liberty to allow peers to decide for themselves whether they participate to the study. Fifthly, *recruitment quotas* (*i*.*e*., the fixed maximum number of names respondents are asked to recruit) have been shown to be effective means for reducing the impact of subjects with large personal networks on the recruitment patterns.

By convention [6], RDS starts from a set of seeds that are financially incentivized to recruit peers. The same system of incentives is applied to all recruits, irrespective of their status—*seeds* or *referrals*. The chain-referral mechanism works only with objective verifiable criteria for assessing membership in the targeted population. Very clear traits for establishing membership are useful for cases of subject duplication (*i*.*e*., multiple participation under different identities) or of subject impersonation (*i*.*e*., cases when a subject pretends to be one of her peers just to collect the incentives). Generally, sampling is completed when either the targeted population is saturated, or a specific size and content of the sample has been reached. Evidently, RDS can be practiced only for populations which exhibit a contact (relational) pattern; there should be ties connecting peers. Furthermore, it is only possible for those cases wherein a trait defining membership in the population is available for objective verification.

In addition to the sampling procedure, [16] advanced an RDS population estimator that accounts for both the differences in homophily across groups and the variation in the size of the personal networks (*i*.*e*., subjects’ number of social contacts). The development of this estimator was critical, as the organization of social (network) structures is generally *homophilous* [19], *i*.*e*., individuals tend to interact with similar others. Consequently, in practice, it was observed that homophily exponentially inflates the standard errors. That was solved by subdividing samples in homophily breakpoints to control for the variability of the estimates.

Heckathorn’s RDS estimator is asymptotically unbiased (*i*.*e*., biases are only of the order of 1/n, where *n* designates the sample size) under the following assumptions [20]: *i*) each subject is connected by at least one link to the rest of the targeted population (*network embeddedness*); *ii*) all members of the targeted population belong to a single *component*, *i*.*e*., every member of the targeted population is part of one global network; *iii*) sampling is performed with replacement (sampling fraction is as small as possible); *iv*) the personal network size is accurately reported by each respondent; *v*) each subject randomly recruits from her network (satisfying this assumption, respondents are inversely weighted by the size of their personal network); *vi*) each subject recruits a fixed number of peers.

Another way of approaching link-tracing designs involves adaptive sampling, *i*.*e*., information collected during the sampling process orients sampling work [21]. Specifically, only respondents who satisfy specific criteria are asked to recruit peers. Estimators from adaptive sampling are valid as long chain-referral waves reach saturation and seeds are randomly selected. This method, which implies maximum likelihood estimation, is limited to instances wherein initial respondents can be randomly drawn and exhaustive link-tracing is feasible in the population.

Other estimators have been developed using egocentric data collected via RDS, *i*.*e*., each respondent, who is connected to her recruiter, provides information on the composition of her personal network or about the proportion of her peers sharing specific attributes [22]. Particularly, this method estimates not only the inclusion probability for every respondent but also for any of her alters or peers. The transition probabilities (*see* [6], for a discussion) are computed based on each respondent’s network composition (alters and their attributes). Using simulations, it was shown that the ego network approach provides estimates for which two important biases were controlled, *i*.*e*., differential recruitment (different patterns of recruitment) and peer underreporting. The main limitation assigned to this method refers to the respondent being able to accurately provide information about the number of alters, which in practice is highly questionable.

#### Link-tracing sampling from transnational social fields

Currently, there is a wide consensus among migration scholars that both migrants and non-migrants’ lives are, to varying degrees, transnational [23]. Cross-border activities and transnational practices, such as communication (via telephones, Skype, WhatsApp, or social media platforms), travel, flow of money and other forms of remittances [24] have been used as indicators of the intensity of transnationality in individuals’ life [12, 25]. As previously pointed out, migrants live multi-sited lives that include not only their home and destination places but other sites worldwide [26]. This way of living connects migrants to other migrants and non-migrants, and, in effect, produces “multiple interlocking networks of social relationships” [27] or *transnational social fields i*.*e*., “networks of networks that stretch across border-states” [28]. Inside these social structures, lives of non-migrants are also transformed despite their immobility [24].

The research on TSFs traditionally tended to disregard the potential benefits of incorporating social network analysis into its methodological apparatus. However, recently, efforts have emerged to represent TSFs through the use of social network analysis tools [10]. Four classes of approaches based on the unit of analysis are identified [5]: *the personal network approach* (focused on individuals), *the household survey approach* (focused on households), *the simultaneous matched samples methodology* (focused on dyads), and *the bi-national link-tracing design* (that encloses a community focus). While the first approach enquires about network members regardless of where they live, it does not sample these network members for further investigation. The other methods, in contrast, tend to invite one or multiple network members of respondents to participate in the research to investigate for example the effect of migration experience of relatives on migration intentions, the transnational exchange of remittances and services, or the configurations of care relationships in transnational families.

The binational link-tracing design [11–13] is heavily built on the simultaneously on-going methodological efforts of transforming chain-referral designs into probability sampling methods [7, 13, 29, 30]. In a nutshell, the binational link-tracing design deploys RDS by sampling individuals both in the sending and receiving places of a migration corridor. Specifically, in the first phase, the elicitation of the TSF starts with a small convenience sample of seeds in the area of destination, after performing ethnographic fieldwork in the community. Individuals in the initial sample nominate other people in the origin and destination places. On the one hand, they are asked to describe their personal network, by eliciting a list of network members (friends, family and acquaintances in the area of origin, the area of destination, returned migrants) and enquiring about their characteristics. Respondents are not asked whether the network members are connected among each other; some of this information should be available through the link tracing network if the sampling fraction is high enough. On the other hand, respondents are asked to give a small number of names of people in both the area of origin and destination who might want to participate in the survey (referrals). The referrals in the destination place are then asked to participate in the survey. This procedure is continued until the desired sample size is reached in the place of destination.

In the second phase, data are collected in the community of origin, based on the referrals of the participants in the destination area. Again, information on their personal networks is elicited. [13] applied this technique to study a migrant community spanning three regions: The Raleigh-Durham-Chapel Hill area of North Carolina; Houston, Texas; and Guanajuato, Mexico, with more than 600 respondents in total.

After data collection, the information is combined to construct a network embedding all the interviewees and their referrals. To do so, it is essential that all individuals are uniquely identified. Therefore, respondents were asked to give the first four letters of the first names and of the surnames of themselves and their network members, without affecting respondent compliance due to privacy concerns. The authors have later also successfully experimented with other identification techniques, namely by using the last four digits of nominees’ phone numbers [11]. Once data belonging to unique individuals are matched, the identifiers can be substituted for others for complete anonymization. Among others, the authors showed that the network underlying the TSF is an important vehicle for opinion formation about migration, and how transnational communication is affected by both individual and network characteristics.

As [5] stress, the resulting network is only a sample or a part of the total TSF; inferences about the whole TSF could subsequently be derived through statistical or mathematical modelling [30]. Moreover, as [13] only asked about people living in the communities of origin and destination, individual transnationality could not be estimated in general, but only with regard to the given corridors. In our study, we grasp the approach introduced by [13], but significantly develop the methodology. The Methods section describes our methodological framework, while emphasizing its distinctive features and commonalities in relation to previous endeavors (*i*.*e*., the work of Mouw and colleagues).

### Romanian migration to Spain

Migration inside the European Union (EU) is highly dynamic [31]. Currently, approximately 20 million European citizens live in a EU country in which they were not born [32]. In 2017, nearly 4.0% of the EU citizens of working age (20–64) were residing in another EU member state–a share which increased from 2.5% in 2007 [33]. Romania is among the *top 20* countries in the world with the largest diaspora populations [34]. Among the EU citizens of working age, Romanians have been the most mobile,–with 19.7% of the population living in another EU member state [33]. As of January 2018, Romanians were estimated to be among the top five most numerous foreign populations in, for instance: Italy (23% of the total foreign population), Spain (15%), Hungary (14%), Slovakia (9%), Portugal (7%) [33]. Since 2000, Romanian migration trajectories with the largest annual increase have been directed towards Italy and Spain [31]. Consequently, it is no surprise that, on January 2018, the Spanish Institute of Statistics registered more than 675,000 Romanian residents in Spain, *i*.*e*., the largest EU foreign population and the second largest foreign population after the Moroccans. In parallel, the Italian National Institute of Statistics reported a tally of more than 1,190,000 Romanian residents in Italy, the largest foreign population.

As already reported [35], Romanians in Spain are geographically unevenly distributed, being concentrated in geographically bounded areas (*i*.*e*., migrant enclaves). One of these Romanian migrant enclaves is established in Castellón and accounts for at least 11% of the total population in this region. Romanians who had firstly arrived in Castellón were predominantly from Dâmbovița [36], a Romanian county with a resident population of 487,115 people (the population of 18 years and older has a size of 399,526, wherein 49% male, 51% female; as of July 2020, Romanian National Institute of Statistics) situated at 78 km North-West of Bucharest, Romania. The steep increase of the migration flux of Romanians to Spain has been underpinned, since 2000, by *institutional factors* (*e*.*g*., recurrent processes of regularization in Spain, free mobility due to Romania adhering to EU since 2007, Spanish immigration policies, aging of Spanish population etc.) and *linguistic proximity*. As a result, many Romanians from Dâmbovița chose Spain as their destination place. As of January 2018, the number of inhabitants of the province of Castellón with Romanian nationality was 38,231, and the number of inhabitants of 18 years and older 30,880 (among the 18+, 47% males, 53% female; average age 40.6 years, *SD* = 12.2; [37]). Romanians rapidly became the nationality with the largest number of residents and with the highest number of employees with a formal contract in this area [36].

## Methods

This section introduces and describes our research design. Specifically, we measured the TSF created by Romanian migrants residing *in* a bounded area in Spain (Castellón), and by their social contacts (relatives, friends and acquaintances) living in Castellón (or other places in Spain), in Dâmbovița (or other places in Romania), and elsewhere in the world. We provide details on the studied population, the sampling procedure and the reward system for participation, on the questionnaire, and the unique identification of individuals. We also explain how various biases (initial sample biases, masking biases, social desirability) were addressed. Additionally, we shed light on the specificity of the current methodology as well as on the commonalities with previous work. The dataset, the code and questionnaire are available for replication [38]. The data collection process had the Catalan Data Protection Authority approval number 217102004-J. Moreover, the participants gave their written consent for data to be anonymously analyzed.

### Sampling and procedures

**Fig 1:**
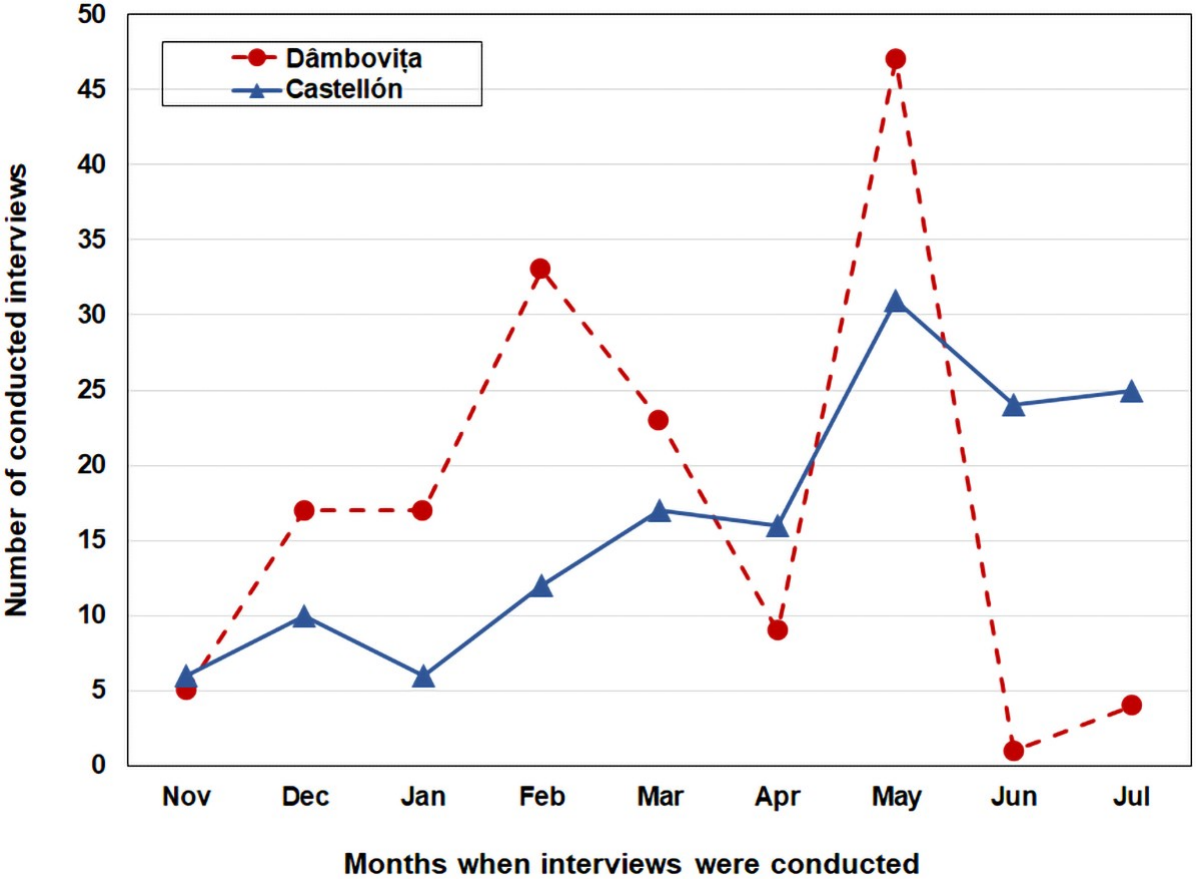
The process of link-tracing data collection, by month and location. The lines are indicating the time variation of the conducted interviews (red line for the Dâmbovița site, while blue for the Castellón site).

Between November 2017 and July 2018, we conducted face-to-face structured questionnaire-based *pen-and-paper personal interviews* with 303 participants in two sites: 149 in Castellón, and 154 in Dâmbovița. Three classes of respondents were sampled: *migrants in Spain* (Romanians living in Castellón, Spain), *return migrants* (Romanians who previously had lived in Castellón, Spain, but returned to Dâmbovița, Romania) and *non-migrants* (people living in Dâmbovița, Romania, who never migrated to Spain). The interviews were conducted, in parallel, by international researchers affiliated to the study. In Dâmbovița (Romania), the fieldwork was undertaken by a team of five scholars who conducted all interviews in Romanian. A team of three scholars of different nationalities undertook the fieldwork in Castellón (Spain), conducting 89 interviews in Romanian and 60 in Spanish. Participants were allowed to freely choose the physical places of their interviews to make them feel comfortable (*e*.*g*., home, pubs, restaurants, public gardens, on the street). Interviews were scheduled using the means suggested in advance by the participants, such as: telephone, WhatsApp or Facebook. After each interview, brief reports were written up by the researchers. The coordination of data collection process as well as solving administrative tasks were carried out through both communication technologies (*e*.*g*., Skype meetings, WhatsApp, Email and Voice Calls) and on-site face-to-face meetings. Fig 1 illustrates the time evolution (in months) of the number of conducted interviews.

We deployed a *link-tracing* sampling design, a procedure essentially based on a chain-referral way of collecting data. Prior to sampling, a year of ethnographic fieldwork research had been conducted in Castellón, which provided important insights on the Romanian migrant population, such as its community structure and social organization, its geographical mapping etc. As previously argued [39], the quality of the sampling from a hidden population is heavily affected by the accuracy and the comprehensiveness of the ethnographic mapping. Consequently, in our case, building on the field reports of the ethnographic research stage, a number of nine *seeds* (the sample of initial subjects, *i*.*e*., Romanian migrants) living in Castellón was *purposively* selected [40].

The seeds were selected to be as *heterogeneous* as possible to ensure that: a) the TSF surrounding the Romanian migrant community in Castellón was widely and extensively explored; b) the chain-referral network linkages did not collapse into a single network component after only a few waves. The heterogeneity of the initial number of seeds was achieved by using relevant and critical demographic features such as *sex*, *marital status*, *age*, *parenthood*, *level of education*, *religion*, and *work status*. These features were indicated as essential for the link-tracing sampling process by the insights of the ethnographic fieldwork undertaken in Castellón. Additionally, the seeds were selected from sub-groups within the Romanian community with little connection between them.

Each of the respondents and the subsequent referrals, after being interviewed, was asked to provide contact details on *three persons* (relatives, friends, and acquaintances) living in Castellón and on *three persons* living in Dâmbovița. Respondents were informed to nominate as referrals only at least 18-year-old people with Romanian nationality. Eligibility for participants recruited in Castellón included residence for at least six months. The data collection process proceeded in a chain-referral way or through *referee–referral* network linkages: from the *seeds* to the *first wave* of respondents, then from the first to the *second wave*, then from the second to the *third wave* etc. until a target sample size was attained.

For ethical reasons, participants to the study received a single rather than a dual monetary incentive for both accepting to be interviewed and helping the research team in recruiting other people. Specifically, respondents (referees) were not only asked to *identify* their peers but also to *recruit* them into the research, to reduce *the masking bias*, as explained before. Members of the research team were allowed to contact the referrals only after receiving the confirmation from the referees. Due to the inexistence of similar previous link-tracing sampling studies in the context of Romanian migration, participation and recruitment rewards were set at ten Euros, in a *face validity* fashion. It was estimated that the amount of money was, on average, sufficiently high to induce participation, and at the same time not too consistent to generate *social desirability effects*, *ethical problems of coerciveness*, or enlisting persons not part of the hidden population. Falsely claiming membership of the study population was controlled and validated by screening both the responses provided to the questionnaire items and the information collected from other participants.

We collected data in the two sites *simultaneously* as we wanted the time between referral and interview to be small to avoid respondents falling out. Identification data on the referrals (peers recruited by already interviewed participants or *referees*) was exchanged by the two research teams in a *ping-pong* game manner. Information, provided by the referees living in Castellón, on referrals living in Dâmbovița, was electronically transmitted by the Castellón *team* to the Dâmbovița *team*, and vice-versa. This electronic transfer of personal contact data was governed by a pre-defined protocol for data anonymization (as a part of a general study Ethics protocol approved by the ethical review board of the Autonomous University of Barcelona). The identification information (full name and contact details, such as phone number, Facebook account or email) was, afterwards, encoded using an alpha-numeric system: the first three letters of the name, the first three letters of the surname and the last four digits of the phone number. Before being interviewed, each participant was informed about the research, asked for his / her contact to participate in the research, and (if consenting) signed a written consent form. If consent was not given, referrals were not interviewed.

We theoretically set a general sample size target of at least 300 interviews, approximately evenly split by the two sites: Castellón and Dâmbovița. We started with fewer seeds and extended the number when we found that six useful referrals were hard to get. Eventually, we needed nine seeds to accomplish a volume of 303 valid interviews (149 interviewees in Spain and 154 in Romania). The impact of the purposively selected sample of seeds on subsequently selected subjects, *i*.*e*., the biases of the non-randomly selected initial seeds, was shown in the literature to be filtered out due to the attained long chains of referee—referrals. In our case, the resulting link-tracing network (*i*.*e*., the network obtained after interconnecting the participants through the link-tracing) has a maximum wave length of 16.

### The questionnaire

We designed and separately applied three customized questionnaires for *migrants in Spain* (people living in Castellón, Spain), *return migrants* (people who previously had lived in Castellón, Spain, but returned to Dâmbovița, Romania) and *non-migrants* (people living in Dâmbovița, Romania, and who never migrated to Spain). Despite their customization, the questionnaires included a core-set of items for all study participants to allow for comparisons across the three groups of subjects.

The questionnaires were devised in English and translated afterwards into Romanian and Spanish. To control the accuracy, validity and the quality of the translation, *forward* and *backward* translations were employed [41]. The pre-final versions of the questionnaires were assessed in an *expert committee* fashion [42] and *pilot-tested* [43] on a sample of five participants. After analyzing their responses, the questionnaire was revised to ensure the validity and reliability of the final version [44].

The questionnaires had several *blocks* of items. The first block registered participant’s identification data, such as *participant’s alias* (to ensure anonymization, participants’ identity was encoded using an alpha-numeric system: first three letters from name, first three letters from the surname and the last four digits of the telephone number), *place of residence*, *sex*, *and date of the interview*. The second, third and fourth blocks enquired respectively about respondents’ attributes (*birth year*, *marital* and *parenthood status*, *level of formal education*, *work status and place*, and *religion)*, *life in Romania* and *migration experience to Spain* (e.g., *work experience*, *decision on migration*, *mobility and migration experience*, *properties owned in Romania and other countries (Spain*, *included)*, *circulation of remittances*, *cultural consumption*, *social identity perceptions*, *satisfaction with life*) and *institutions (organizations) currently supporting respondents’ migration experience* (if applicable).

In the fifth block, respondents were asked to elicit a specific number of personal contacts (relatives, friends and acquaintances) they knew in different places, including their closest contacts; the so-call *name generators* allowing the construction of a personal network for each participant. Precisely, respondents were asked to elicit: a) maximum ten friends and acquaintances living in the current place of residence (Castellón for migrants and Dâmbovița for non-migrants and returnees); b) maximum five relatives living in *the current place of residence*; c) maximum five relatives, friends and acquaintances who had lived in Castellón but now live in Romania; d) maximum five relatives and five friends and acquaintances who live in the other place of the TSF (Dâmbovița for migrants and Castellón for non-migrants and returnees); e) maximum five relatives and five friends and acquaintances living in other places than Castellón and Dâmbovița. The application of the five name generators could theoretically elicit a maximum of 40 network members (“alters” in personal network research): 15–20 relatives and 20–25 friends and acquaintances. Additional questions were used to collect information on the elicited alters (these questions are called “name interpreters” [45], such as their attributes: *sex*, *occupation* and *religion*, and the respondent’s relationship with him/her, such as: *the nature of the relationship* (*e*.*g*., workmates), *duration* (in years), *emotional closeness*, and *communication frequency*. In the last section of the network module, we measured relationships among network members. In line with the suggestions available in the literature for reducing *respondent burden* in such questions [46–48], we randomly sampled nine alters from those originally elicited to measure network structure. Respondents were asked to mention, for each pair of sampled alters, whether they *knew each other and could contact each other independently of the respondent*.

In the last block of the questionnaire, respondents were requested to recruit referrals eligible to participate in the research (*i*.*e*., Romanians living in any of the two fieldwork sites over the age of 18-year old), and to ask them, either on the spot or after the interview, whether they would be willing to participate in the research. We solicited for maximally three individuals per fieldwork site. In some instances, the recruited people had been also nominated as a network member in *block 5*.

### Interconnecting personal networks and the structure of the data

To build a single, *multi-layered network* or, in other words, the *network of networks* (or *a representation of the TSF*; the interconnecting of the link-tracing participants as well as their nominees, *i*.*e*. referrals and alters), we uniquely identified each individual in the research, taking into account that an individual could appear multiple times in the data (*e*.*g*., as a network alter of one respondent, a referral of another respondent and ultimately as respondent). The identification implied an extremely cumbersome and tedious procedure. Firstly, all nodes received an alphanumerical code in the data collection process. As indicated before, alphanumerical coding was derived from the first three letters of the name, first three letters of the surname and from the last four digits of the phone number. In some cases, due to missing data (*e*.*g*., the alters for whom egos were not able to provide a phone number, or a last name), special coding was assigned. Secondly, the allocated alphanumerical codes were subject to a data cleaning and validation process. The process was meant to detect and correct inaccurate records either due to data entry errors or conflicting coding (*e*.*g*., in some cases, two different nodes were assigned the same code, whereas in others, the same node was allocated different alphanumerical codes). The data cleaning process was conducted using various methods: from manual screening and the use of the *RecordLinkage R package* [49] in the initial stages, to employing Microsoft *Excel VLookUp* function. Matching individuals with unique alphanumerical codes was validated by examining additional identifying information such as: *sex*, *occupation*, *place of residence*, *religion*.

**Fig 2:**
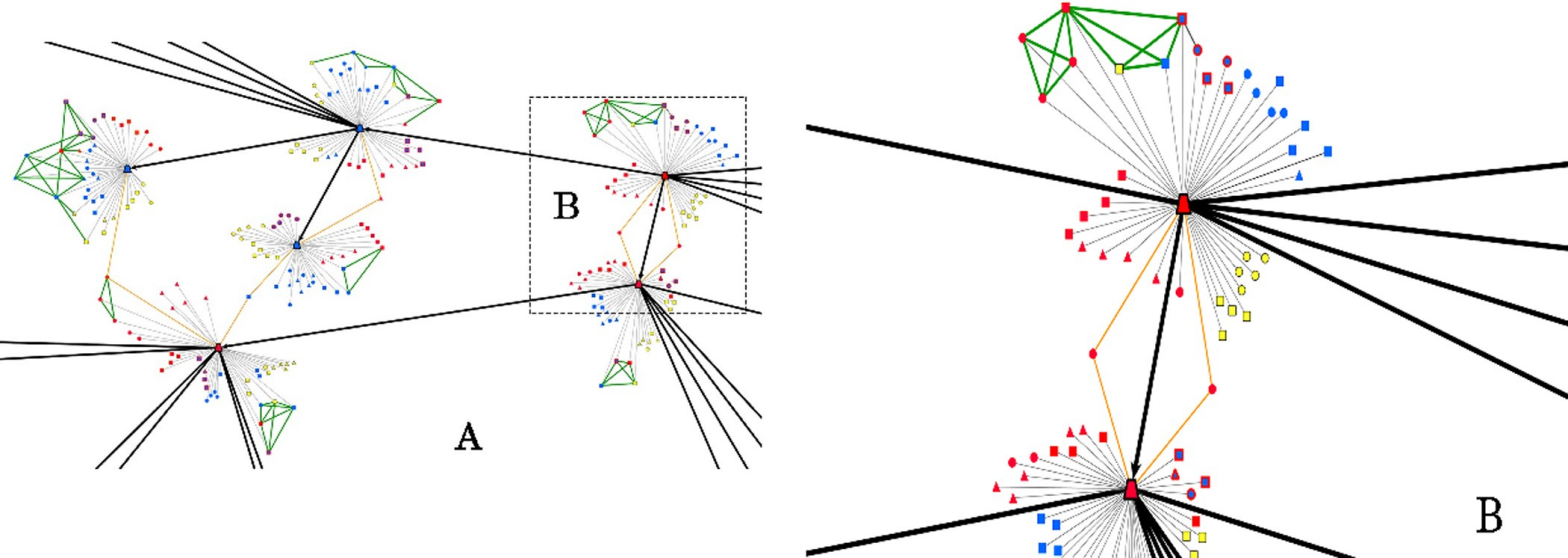
Multi-layered network (*the network of networks*) built by the link-tracing sampling method. (A) Inter-connected personal networks, through link-tracing ties and shared alters. (B) Zoom in on the personal network of respondents (the dotted area of A in the panel). Node colors designate places (countries) wherein individuals currently live, *i*.*e*., ‘blue’ for Dâmbovița (Romania), ‘red” for Castellón (Spain), ‘yellow’ for *other places* than Dâmbovița and Castellón (these *other places* could be other regions in Romania and Spain, or even other countries–for simplicity and illustrative purposes, in this example, we decided, by ‘yellow’, to mark other countries. Node shapes designate classes of individuals (alters) elicited by the fixed-number alter name-generators: ‘squares’ designate ego’s *family members*, ‘triangles’ designate ego’s *friends*, while ‘circles’ designate ego’s *acquaintances*. The ego (the respondent) is marked by a trapezoid shaped—black bordered node. Ego’s alters (family members, friends and acquaintances) who lived in Castellón (Spain) and returned to live in Romania are marked by ‘red’ bordered shapes (*e*.*g*., returned family members are marked by blue squared nodes with red border). A sub-set of nine alters was randomly sampled from the elicited set of alters, and the alter-alter existing ties represented. This is illustrated by the green edges. If two nodes are connected by an edge, that means the two nodes know each other and can contact each other independently from the ego. As all the nodes represent ego’s alters, the respondent (the trapezoid shaped node) is connected to everybody through a tie (see the thin gray edges). Orange edges indicate the cases wherein two egos share alters. The black arrows indicate the direction of the link-tracing sampling: an arrow’s origin marks the referee while the head of the arrow marks the referral. The plots were built using visone [57].

The unique identification of individuals allowed us to interconnect the data from different respondents to build a multi-layered network. As illustrated in Fig 2, firstly, we generated *the personal network* of each of the 303 participants in the study. Each respondent (ego) is marked by a *trapezoid-shaped*, *black-bordered* node. Node color represents the place of living: “red” for Castellón (Spain), or “blue” for Dâmbovița (Romania)–*see* Fig 2A and 2B. Each ego is embedded in a personal network comprising a maximum number of 40 alters. Alters are designated by nodes of variant shapes and colors based on their corresponding type. Particularly, relatives are indicated by *squares*, friends by *triangles*, and acquaintances by *circles*. Colors again mark places of residence: “red” for Castellón (Spain), “blue” for Dâmbovița (Romania), “yellow” for other places (or countries) than Castellón or Dâmbovița. Ego’s alters (relatives, friends and acquaintances) who had lived in Castellón and returned to live in Romania are marked by blue nodes with ‘red’ borders (*e*.*g*., returned relatives are marked by blue squared nodes with red borders). Additionally, from the set of elicited alters a sub-set was randomly sampled to ask respondents, for each pair, whether these persons knew each other. The ties among these alters are marked by green edges.

In a second step, all of the 303 personal networks were interconnected through the link-tracing referrals. This is illustrated in Fig 2 by the black thick arrows. By *zooming in* into the visualization exhibited in Fig 2A and 2B allows for a quick inspection of the interconnecting procedure. Each arrow has the referee as origin, while the arrow-head indicates the referral. Where applicable, the respondents’ personal networks were also interconnected through their shared alters (common social contacts). Orange edges are indicative of alters shared by multiple respondents.

The sample from the TSF or the *network of networks* (the network generated by interconnecting personal networks, *see* Fig 2) indicates a multi-layered data structure. The first layer consists of 303 *personal networks* that can be independently analyzed, both in terms of composition and structural features. The second layer includes *the link-tracing network*, *i*.*e*., the network that illustrates the implementation of the link-tracing sampling method. This network consists of 1,068 nodes and 1,187 ties. The third layer results from interconnecting personal networks (*the network of networks* that includes 4,855 nodes, 5,477 directed ties and 2,540 edges).

### The specificity of the ORBITS methodology

In this sub-section, we present the specificity of our current methodology (ORBITS) as well as the commonalities with studies [12, 13, 25] built on a similar binational link-tracing approach (*i*.*e*., *the Network Survey of Immigration and Transnationalism*, the *NSIT study*).

Firstly, the ORBITS study employed a simultaneously multi-sited data collection process, whereas the NSIT study collected the information in two stages (research in the destination places of Mexican migrants preceded research in their origin place). Simultaneously collecting data on both sites was deemed to enable a timely follow-up of migrants and non-migrants. Secondly, the NSIT outsourced the data collection to community members while the ORBITS data was collected by the research team. Given the complexity of the questionnaire and of the participant selection procedure, in-house data collection increases the validity of the acquired information. The ORBITS study has two unique features. First, we collected information on the ties between the personal contacts (alters) of the participants, *i*.*e*., alter- alter edges, for a random selection of 40 edges. This is an important advancement as it provides insights on the structure of the participants’ social life and it estimates network structures more adequately. For instance, it allows the investigation of the composition and structural characteristics of the participants’ surrounding contacts. Second, we asked respondents to also nominate people in other places than Castellón or Dâmbovița. This piece of information is crucial for unveiling participants’ connections to other places in the two countries and to other countries worldwide, and not to bi-locally limit transnationalism. We would conclude that, in comparison to the NSIT study, the ORBITS research design permitted the collection of a richer information and ensured a higher level of data quality control.

Table 1 briefly illustrates the methodological differences between the two studies. In terms of commonalities, both studies: a) share the same research approach (binational link-tracing sampling from TSFs), b) collect data from the destination and the origin places of migrants (community-oriented procedure), c) collect cross-sectional data, and d) use demographic variables to uniquely identify TSF members, *i*.*e*. participants to the research as well as their nominees, either referrals or alters (as a way of ensuring both confidentiality and anonymity).

**Table 1:** Methodological differences between NSIT and ORBITS studies.

Dimension	NSIT study	ORBITS study
Size of samples	Origin place: Guanajuato (Mexico), n = 410	Origin place: Dâmbovița (Romania), n = 154
	Destination places: North Carolina (US), n = 146 and Houston (US), n = 51	Destination place: Castellón (Spain), n = 149
The design of the data collection process	*Two-steps*: firstly, in the origin places, afterwards, in the destination.	Link-tracing sampling design: nine seeds (Castellón).
	(a) link-tracing sampling design, in the destination places: 12 seeds (North Carolina) & five seeds (Houston). The collection of data through link-tracing sampling was limited to the population of interest living in the destination places.	From the nine seeds, the data collection process went on, being employed a simultaneously multi-sited data collection process in the destination and origin places.
		Specifically, in the first wave, each seed was asked to recruit three referrals living in the origin and three, in the destination.
	(b) “*pyramid selection approach*” [12] or 4-level collection strategy in the origin place. 20 seeds (Guanajuato) were randomly selected from the pool of alters of all respondents elicited in the first step (in the destination). On the 2-level, two friends and two relatives of each of the seeds were interviewed. On the 3-level, one friend and one relative of each of the participants on the 2-level were interviewed. On the 4-level, for each of the participants on the 3-level, one either friend or relative, randomly selected, was interviewed.	
		On the second wave, both the three referrals in the origin and the three referrals in the destination were asked to recruit three people in the origin and three in the destination.
		Subsequently, on additional waves, referrals nominated by the referees interviewed in previous waves were also contacted and interviewed. The process halted when the sample size target was reached (at least 300 interviews).
Data collection	Community members collected the data, aside pretests.	Members of the ORBITS study team collected the data and conducted the pretests. ORBITS researchers simultaneously coordinated in a “*ping-pong*” fashion across the two sites.
Name generator	*Destination places*: ≤10 friends and acquaintances & ≤ 6 relatives (living in the destination), ≤ 6 relatives / friends and acquaintances (living in the origin) and ≤ 5 returned migrants.	*Destination place*: ≤10 friends and acquaintances & ≤ 5 relatives, living in the destination; ≤ 5 friends, acquaintances or relatives returned to Romania; ≤ 5 relatives & ≤5 friends and acquaintances living in the origin; ≤5 friends and acquaintances & ≤5 relatives living in other places (than the origin and the destination).
	*Origin place*: ≤ 6 friends and acquaintances / relatives (living in the origin) and ≤ 6 friends and acquaintances / relatives living in the destination places.	*Origin place*: ≤10 friends and acquaintances & ≤ 5 relatives, living in the origin; ≤ 5 friends, acquaintances & relatives returned to Romania; ≤ 5 relatives & ≤5 friends and acquaintances living in the destination; ≤5 friends & ≤5 relatives and acquaintances living in other places (than the origin and the destination).
	Maximum unique number of elicited alters: 27. [12, 13]	Maximum unique number of elicited alters: 40.
Alter-alter edges	Not measured	To avoid respondent burden, a sample of nine alters is randomly sampled from the pool of elicited alters by each participant. Subsequently, the alter-alter ties are measured, as existent or non-existent, according to the participants’ perceptions.

## Results

### Demographics of participants and refusals

Despite the demographic heterogeneity of the seeds (Table 2), the initial sample was composed of individuals whose living experience in Castellón was consistent (on average they had been living in Castellón for 17 years). This was indicative of their high-level of embeddedness in the local (Castellón) community as well as in the Romanian collectivity living in Castellón. This degree of embeddedness was deemed essential for starting and ensuring the success of the link-tracing sampling procedure.

**Table 2:** The demographic profile of the initial subjects in the sample (the seeds).

*Initial seeds (sample)*	9
*Sex*	
Males	5
Females	4
*Marital status*	
Married	5
Divorced & single	1
Widow(er) & single	1
Single	2
*Age*	
Mean (SD)	44.4 (11.7)
Min (Max)	27 (59)
*Level of formal education*	
Ten years completed with diploma	1
High school (with diploma)	2
Post high school education	2
Higher education (BA degree)	4
*Religion*	
Orthodox	6
Pentecostal	1
Adventist	1
No religion	1
*Work status*	
Employed	8
Unemployed	1

The link-tracing sampling method started from these nine seeds and continued, one wave after another, with a pile of 294 additional interviewees. The data collection process was stopped after the target sample size of at least 300 interviews had been reached (in our case, the process halted at 303 interviews). In the study, 1,059 referrals had been nominated in the two sites (not counting here the initial sample of nine seeds). As a general remark, respondents tended to nominate more women (59%) than men (41%) (χ^2^(1) = 28,61, *p <* .001). Additionally, in Castellón, 67% of all referrals were females while in Dâmbovița, 51%.

Out of the total of 1,068 nodes comprising the link-tracing network, 765 people refused to participate to the study (72%) while 12 interviewees did not provide any referrals. It follows that the link-tracing sampling procedure had a success rate of nearly 28%.

Table 3 reports the distributions of refusals and participants, split by referee’s gender and country of residence (place of living). The number of people who were contacted by the referees and their response rate is about the same in Dâmbovița (539 and 29%, respectively) as in Castellón (529 and 28%, respectively). In addition, in Dâmbovița, the participation to the study is roughly gender-balanced (53% males interviewed), compared to Castellón, wherein more than two thirds of participants were females (72%).

**Table 3:** Distribution of participation by referee’s gender and place of living.

*Referees’ place of living and gender*	*Participated in the research*?		
	Yes	No	Total
Dâmbovița (Romania)			
Males	83 (31%)	180 (69%)	262 (100%)
	53%	47%	49%
Females	73 (26%)	203 (74%)	275 (100%)
	47%	53%	51%
Total	156 (29%)	383 (71%)	539 (100%)
	100%	100%	100%
Castellón (Spain)			
Males	40 (23%)	133 (77%)	173 (100%)
	28%	35%	33%
Females	107 (30%)	249 (70%)	356 (100%)
	72%	65%	67%
Total	147 (28%)	382 (72%)	529 (100%)
	100%	100%	100%
*Total (both sites)*			
Males	123 (28%)	313 (72%)	436 (100%)
	40%	41%	41%
Females	180 (28%)	452(72%)	632 (100%)
	60%	59%	59%
Grand total	303 (28%)	765 (72%)	1,068 (100%)
	100%	100%	100%

The valid percentages are computed both column and row-wise; these may add up to more than 100% due to rounding off numbers.

The distributions for the main demographic characteristics of the survey respondents to the ORBITS study can be inspected in Table 4. In Dâmbovița, 88% *never migrated to Spain*, while the average age within the site is *37-year-old*. Moreover, most of the participants in Dâmbovița were at least *high-school graduates* (36%) and *orthodox* (97%). In terms of *civil status*, *work status* and *parenthood*, the data indicate rather bi-modal distributions, *i*.*e*., *married* or *single*, *employed* or *inactive (students)*. In the place of destination (Castellón), the great majority of the interviewees were *employed* (59%; a much larger percentage than in Romania), *orthodox* (82%, fewer than in Romania), *parents* (69%, more than in Romania) and *high-school graduates or higher* (51%). They were *on average 44 years old*. Based on our ethnographic fieldwork, we believe that these characteristics, except gender (see also the Introduction), represent the Romanian community in Castellón quite realistically.

**Table 4:** Major demographic characteristics of the link-tracing network participants (survey respondents).

	Dâmbovița (Romania)	Castellón (Spain)	*Grand total*
	*place of origin*	*place of destination*	
*Respondents*	156 (100%)	147 (100%)	303 (100%)
*Type of participants*			
Non-migrants	138 (88%)	0 (0%)	138 (46%)
Return migrants	18 (12%)	0 (0%)	18 (6%)
Migrants in Spain	0 (0%)	147 (100%)	147 (48%)
*Sex*			
Female	73 (47%)	107 (72%)	180 (60%)
Male	83 (53%)	40 (28%)	123 (40%)
*Civil status*			
Married	58 (37%)	66 (45%)	124 (41%)
Single (never married)	57 (37%)	36 (25%)	93 (31%)
Unmarried & in a stable relationship	26 (17%)	8 (5%)	34 (11%)
Divorced & single	8 (5%)	25 (17%)	33 (11%)
Widow(er) & single	7 (5%)	9 (6%)	16 (5%)
Separated & single	0 (0%)	1 (1%)	1 (0%)
Other	0 (0%)	2 (1%)	2 (1%)
*Age total*			
Mean (SD)	37.2 (17.0)	43,5 (13.5)	40,2 (15.7)
	(*n = 156*)	(*n = 146*)	(*n = 302*)
Min (Max)	19 (76)	20 (73)	19 (76)
*Age Females*			
Mean (SD)	41.7 (18.6)	44,4 (13.7)	43,3 (15.9)
	(*n = 73*)	(*n = 106*)	(*n = 179)*
Min (Max)	19 (76)	21 (73)	19 (76)
*Age Males*			
Mean (SD)	33.2 (14.4)	41,1 (12.6)	35,8 (14.3)
	(*n = 83*)	(*n =* 40)	(*n = 123*)
Min (Max)	19 (72)	20 (63)	19 (72)
*Level of formal education*			
No formal education	0 (0%)	1 (1%)	1 (0%)
Less than four years	0 (0%)	0 (0%)	0 (0%)
Four years completed	0 (0%)	0 (0%)	0 (0%)
Between five and eight years	3 (2%)	1 (1%)	4 (1%)
Eight years completed with certificate	25 (16%)	7 (5%)	32 (11%)
Ten years completed with diploma	12 (8%)	32 (22%)	44 (15%)
High school (without diploma)	24 (15%)	14 (10%)	38 (13%)
High school (with diploma)	56 (36%)	50 (35%)	106 (35%)
Post high school education	10 (6%)	19 (13%)	29 (10%)
Higher education (BA degree)	19 (12%)	20 (14%)	39 (13%)
Higher education (MA degree, PhD etc.)	7 (5%)	1 (1%)	8 (3%)
*Work status*			
Employed	59 (38%)	86 (59%)	145 (48%)
Self-employed	10 (7%)	10 (7%)	20 (7%)
Unemployed	1 (1%)	18 (12%)	19 (6%)
Retired	17 (11%)	9 (6%)	26 (9%)
Student	54 (35%)	7 (5%)	61 (20%)
Inactive	14 (9%)	7 (5%)	21 (7%)
Other	0 (0%)	10 (7%)	10 (3%)
*Religion*			
Orthodox	151 (97%)	119 (82%)	270 (90%)
Reformed	0 (0%)	0 (0%)	0 (0%)
Pentecostal	1 (1%)	4 (3%)	5 (2%)
Baptist	0 (0%)	7 (5%)	7 (2%)
Adventist	0 (0%)	5 (3%)	5 (2%)
Catholic	0 (0%)	0 (0%)	0 (0%)
Other	2 (1%)	0 (0%)	2 (1%)
No religion	2 (1%)	10 (7%)	12 (4%)
*Do you have children*?			
Yes	73 (47%)	101 (69%)	174 (57%)
No	83 (53%)	46 (31%)	129 (43%)

The valid percentages are computed column-wise; columns may add up more than 100% due to rounding off numbers.

### The link-tracing network

**Fig 3:**
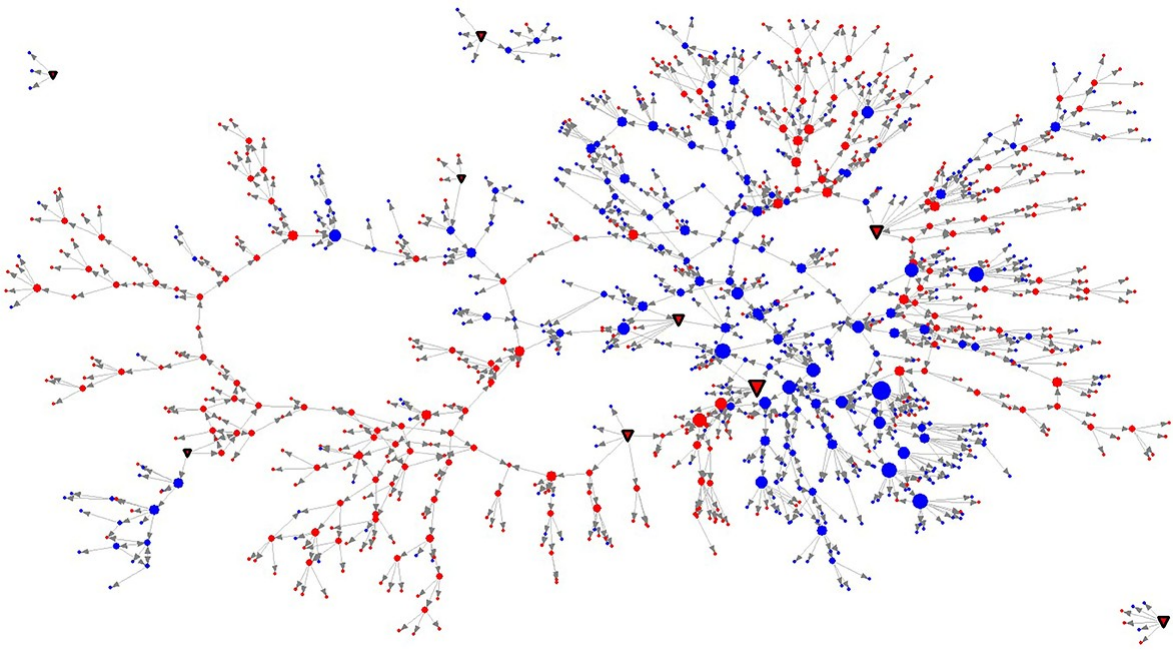
Hairball visualization of the link-tracing network. Node colors indicate three classes of nodes: *red down-triangles*–seeds (individuals living in Castellón, Spain), *red*–people interviewed in Castellón, Spain, *blue–*people interviewed in Dâmbovița, Romania. The network is directed indicating the chain-referral structure of the relational data. Directed dyads are indicative of *referees* (the origin of the arrow) and referrals (indicated by the arrow head). Size nodes are proportional to their out-degree (the number of referrals provided). By dotted perimeters are indicated components (disconnected parts of the network). The plot was built using the UCINET 6.0.

The link-tracing network (the network embedding 303 participants and another 765 referrals who did not participate to the study) consists of 1,068 nodes and 1,187 ties (see Fig 3**)**. The hairball network allows for the inspection of the bi-national characteristic of the employed sampling design. Particularly, there are nine seeds (*red-colored down-triangles*) and 138 nodes (*red-colored circles)* that represent respondents living in Castellón. Additionally, *the blue-colored circles* represent interviewees in Dâmbovița. The pattern of providing referrals (the out-degree) is shown by proportionally increasing the size of each node. Supplementary, the direction of the link-tracing sampling is marked by directed ties.

The network shows that six of the nine seeds, even though they are not directly connected among each other, are embedded in the same *component* (a connected graph wherein all pairs of nodes are reachable through a succession of ties). Within this main component (accounting for 96% of all nodes), paths from some nodes to others are very large (with lengths up to 18). Given small world theories, it is very likely that increasing the sample volume would have resulted in having all nodes in only one component. Also, Fig 3 illustrates node clustering by residence. Namely, in some chains, referrals to people in the other fieldwork site were either not given or they were not willing to participate. The total 1,187 referee-referral arcs of the link-tracing network have the following distribution, within and across sites: Dâmbovița–Dâmbovița (*n = 421*), Castellón–Castellón (*n = 506*), Dâmbovița–Castellón (*n = 97*) and Castellón–Dâmbovița (*163*).

**Referral pattern by sex and residence in the link-tracing network: Homophily.** For all the referees and referrals in the link-tracing network (even for unsuccessful referrals), we had information about *sex* and *place of residence*. This information allows us to assess whether patterns of homophily can be identified. Specifically, on one hand, we could examine *intra-place* nominations (the tendency of participants to nominate referrals within their own place of residence) and *inter-place* nominations (the tendency toward cross-nomination by place of residence, *e*.*g*., participants in Castellón tend to rather nominate referrals in Dâmbovița). On the other hand, we could inspect whether there are intra- or inter-sex nominations (*e*.*g*., males tend to nominate males or males tend to nominate females). One way to explore response patterns is to work with the observed scores, without performing any further adjustments and manipulations. This naïve approach [11] is suggested here only for illustrative purposes.

**Fig 4:**
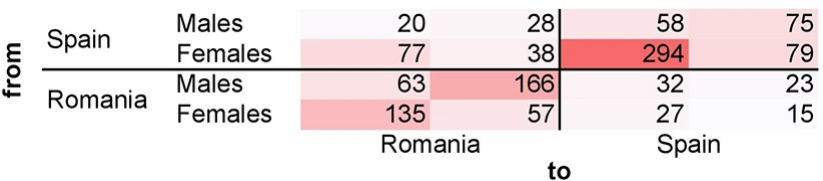
Pattern of referral nominations based on sex and residence. The visualization illustrates the pattern of referral nominations, based on sex and place of residence. For instance, the female respondents living in Romania (Dâmbovița) (see the “from” axis) indicated as referrals (see the “to” axis): 135 and 57 males living in Romania (Dâmbovița), 27 females and 15 males living in Spain (Castellón).

Fig 4 shows the distribution of nominations over *sex* and *place of residence*. *Homophily* and *heterophily sex effects* are indicated both in association with the place of residence, and independently. The color of each cell varies in intensity as a function of the frequency of nominations. Fig 4 illustrates a census of all the nominations. The four-by-four matrix is a mix of sex and place of residence. It indicates, for instance, the nomination tendency of Romanian females; the rows of the matrix are the referees, while the columns are the referrals. The two-by-two matrix allows for independently inspecting male and female-homophily effect. We found that: a) there is an overall tendency toward male- and female-homophily; b) the sex nomination pattern is affected by the place of residence, *e*.*g*., male respondents tend to rather nominate more males living in their proximity than in the other site (that also holds for female nomination pattern).

**Fig 5:**
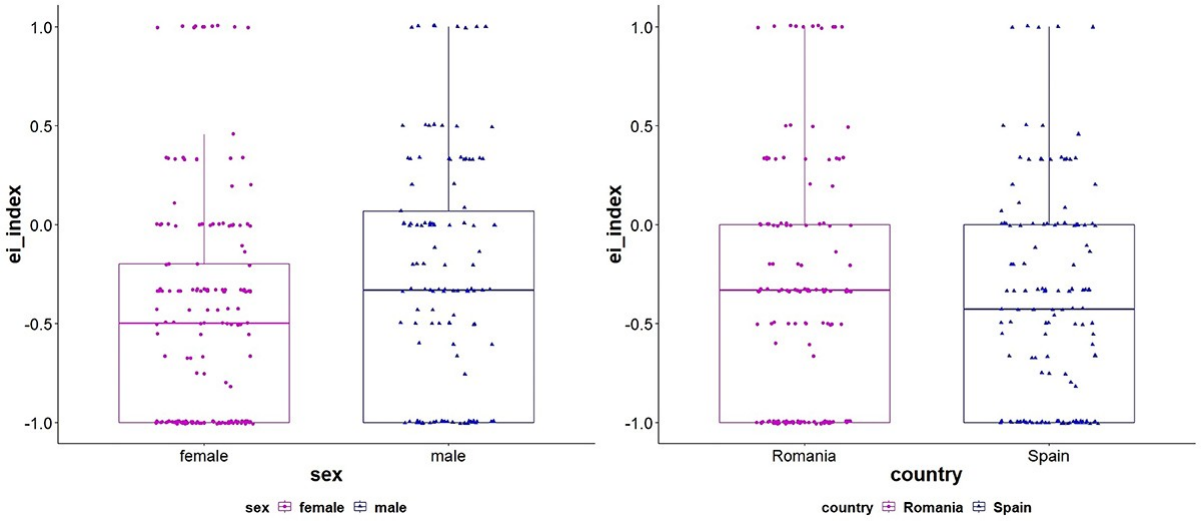
Boxplots illustrating individual distributions of EI-index scores split by sex and residence. For every study participant who provided referrals, an E-I index score was computed, either taking into account sex or place of residence. E-I index is computed by a simple formula: for a specific participant the difference between the number of ties between groups and within group is divided up to the sum of ties (e.g., for a female participant, the difference between her ties sent to males and her ties sent to other females is divided up to the total number of ties).

To show individual variation in the homophily, Fig 5 illustrates the univariate distribution of E-I index [50] scores computed independently for sex and place of residence, in UCINET 6.0 [51]. The index ranges from -1.0 to +1.0 where positive scores indicate heterophily (*e*.*g*., the tendency of males referring to females, or of people in Dâmbovița to people in Castellón). Negative E-I index scores indicate homophily. Fig 5 shows that throughout the link-tracing network there are both sex and place of residence tendencies toward homophily.

Working with the observed values (frequencies) for detecting homophily has two potential validity threats [11]: nominations are conditioned by the relative number of people in each category (*e*.*g*., number of females versus number of males, respondents living in Dâmbovița versus respondents living in Castellón) and by the volume of referrals (*i*.*e*., the number of referrals variates across referees). To manage these potential validity threats, we performed permutation tests to assess whether the observed link-tracing network E-I index score on a specific attribute (sex and residence) is significantly higher or lower than the score expected by chance. According to the results reported in Table 5, the *sex* and *place of residence* uniform homophily effects still hold.

**Table 5:** Sex / place of residence E-I index scores computed in the link-tracing network.

	E-I index scores		
	Expected	*SD*	Observed
Sex	-0.033	0.031	-0.391*
Residence	0.001	0.029	-0.561*

The scores were computed using the *E-I* index routine available in UCINET 6.0 [51]. Both observed E-I index scores on *sex* and *residence* were statistically significant (*p* < .05). The expected *E-I* index scores were computed after conducting 5,000 permutations. The computations took into account the link-tracing size (n = 1,068) and a total edge number of 1,187.

We also used Exponential Random Graph Modeling (ERGM), *i*.*e*., statistical models that allow for explaining tie patterning in social networks. For instance, tie formation processes within social networks can be accounted for by looking at: a) various local network configurations (*e*.*g*., transitivity structures—*friends of friends are friends*, reciprocity—*you scratch my back and I scratch yours*, preferential attachment, etc.); b) actor attributes *or* node covariates (*e*.*g*., homophily); c) dyadic covariates (geographical distance between persons) [52].

To explain the processes giving rise to the observed link-tracing network, we built several ERGM models [53] for the network of respondents (n = 303). We assessed several determinants of the nomination tie pattern within the link-tracing network of participants. Specifically, we looked at the propensity of people to make nominations in general (*sociality*), and to make nominations based on attributes such as residence and sex (*assortative mixing*). Additionally, we assessed various structural parameters to account for network self-formation processes: the prevalence of actors who make or receive two nominations, the popularity (*geometrically weighted in-degree distribution*) and activity spread (*geometrically weighted out-degree distribution*), tendencies toward triangulation (*geometrically weighted edgewise shared partner distribution*, *i*.*e*., the formation of clusters of triangles) or toward sharing a partner irrespective of whether two participants are connected or not (*geometrically weighted dyad-wise shared partner distribution*) [54–56]. The models were fit on the link-tracing network of study participants (*n* = 303).

Table 6 displays the results of the fitted ERGM models, wherein the estimates indicate the conditional log-odds of any tie occurring in the link-tracing network of participants. In all models, the sociality coefficient (*edges*) is negative and statistically significant, indicating that the number of observed edges is lower than expected by chance alone. This is due to the study design. Moreover, homophily effects are statistically significant (*p <*0.001) throughout the fitted models. As shown in Table 6, males tend to nominate males whereas females tend to nominate females. Additionally, respondents tend to nominate individuals in their place of residence. Model 2 tests for an *age* oriented homophily (the tendency of participants to rather nominate similar peers in terms of the age), while controlling for the other homophily effects (sex and residence). Evidence suggests that an age effect is not present in the link-tracing network (*Est*. *= -*0.00, *S*.*E*. *=* 0.00, *p >* 0.05). Model 3 indicates that out-degree scores of two are not prevalent (*Est*. *=* 0.07, *S*.*E*. = 0.05, *p* > 0.05). Conversely, the number of participants receiving two nominations is less than expected by chance alone (*Est*. *=* -2.35, *S*.*E*. = 0.24, *p* < 0.001). Models 4–6 are informative about the overall organization of edges in the network. First, we notice that a small number of nodes tend to be more popular (*Est*. *=* 5.24, *S*.*E*. = 0.52, *p* < 0.001). Second, in terms of activity, the number of transmitted nominations is not statistically significant (*Est*. *=* -0.07, *S*.*E*. = 0.20, *p >* 0.05). Put it differently, what we observe is not different (*p >* 0.05) from what we should obtain by chance alone. Triadic closure is detected in Model 5 (*Est*. = 2.24, *S*.*E*. *=* 0.11, *p* < 0.001). The number of triangle configurations is higher than the one observed in a random network. The negative estimate of the configurations accounting for *shared partners* (Model 5, *Est*. = -0.11, *S*.*E*. = 0.04, *p* < 0.05) marks that 2-paths (*i* ->*j* -> *k*) tend to close in the observed network. Model 6 is the full model with all the predictors of interest being tested. This model has the best overall fit (AIC = 4251.61, BIC = 4345.72). Predictors that were statistically significant in the previous models still hold their contribution to the formation of the network. Precisely, nominations are patterned by sex (*Est*. = 0.78, *S*.*E*. = 0.14, *p <* 0.001, *Est*. *=* 0.48, *S*.*E*. = 0.14, *p <* 0.001, for male-male and female-female nominations, respectively) and residence (*Est*. = 1.23, *S*.*E*. = 0.17, *p <* 0.001, *Est*. *=* 1.95, *S*.*E*. = 0.18, *p <* 0.001, for Dâmbovița—Dâmbovița and Castellón—Castellón nominations, respectively). Further, evidence does not support the presence of age homophily (*p>*0.05). Referring to the overall structure of the network, we notice triadic closure (*Est*. = 3.11, *S*.*E*. = 0.16, *p <* 0.001) and a tendency of 2-path configurations to eventually close (*Est*. = -0.23, *S*.*E*. = 0.10, *p <* 0.05). Also, a small number of participants receive more nominations (are more popular) in comparison to the rest of the network (*Est*. = 5.83, *S*.*E*. = 0.55, *p <* 0.001), whereas nodes are comparable in terms of the number of the recommendations made (activity) (*Est*. = 0.33, *S*.*E*. = 0.20, *p >* 0.05). In Table 6, the coefficients of the predictors (*Est*.) indicate the conditional log-odds of a tie connecting two participants. Goodness of fit statistics for the ERGM fitted models are available for consultation in [38].

**Table 6:** Micro-level determinants of nomination tie patterns, in the link-tracing network of study participants.

	Model 1	Model 2	Model 3	Model 4	Model 5	Model 6
edges	-7.01 ***(0.15)	-6.96 ***(0.16)	-5.02 ***(0.27)	-8.84 ***(0.24)	-6.78 ***(0.17)	-8.71 *** (0.33)
Nodematch (sex homophily: male–male)	0.79 ***(0.14)	0.78 ***(0.14)	0.83 ***(0.15)	0.84 ***(0.16)	0.72 ***(0.12)	0.78 *** (0.14)
Nodematch (sex homophily: female–female)	0.51 ***(0.12)	0.52 ***(0.12)	0.50 ***(0.14)	0.49 ***(0.14)	0.47 ***(0.11)	0.48 ***(0.14)
Nodematch (residence homophily: Dâmbovița–Dâmbovița)	1.54 ***(0.16)	1.54 ***(0.16)	1.38 ***(0.17)	1.34 ***(0.17)	1.41 ***(0.15)	1.23 ***(0.17)
Nodematch (residence homophily: Castellón–Castellón)	1.87 ***(0.15)	1.88 ***(0.15)	2.09 ***(0.18)	2.15 ***(0.19)	1.69 ***(0.15)	1.95 ***(0.18)
nodecov.age (age homophily)		-0.00 (0.00)				-0.00 (0.00)
ostar2 (two nominations made)			0.07 (0.05)			
istar2 (two nominations received)			-2.35 ***(0.24)			
gwodeg.fixed.0.25 (activity)				-0.07 (0.20)		0.33 (0.20)
gwideg.fixed.0.25 (popularity)				5.24 ***(0.52)		5.83 ***(0.55)
gwesp.fixed.0.25 (triad closure)					2.24 ***(0.11)	3.11 ***(0.16)
gwdsp.fixed.0.25 (shared partners)					-0.11 * (0.04)	-0.23 * (0.10)
AIC	4695.24	4695.25	4500.66	4435.55	4578.55	4251.61
BIC	4742.29	4751.72	4566.54	4501.42	4644.43	4345.72
Log Likelihood	-2342.62	-2341.63	-2243.33	-2210.77	-2282.28	-2115.81

*** *p* < .000,

** *p <* .001,

*p < .01. The ERGMs were estimated using the statnet suite [52]. Standard Errors are reported in parentheses.

In sum, the results of this section show that the referral patterns show clear pattern of homophily in sex and place of residence, but not in age. This shows us the importance of seeds being diverse in sex and place of residence, or of using adapting sampling, i.e. not interview all referrals willing to participate but use additional qualifiers to obtain a balance sample.

#### The link-tracing network chains

The link-tracing network can be decomposed into nine referee-referral chains (both participants and non-participants) stemming from each of the nine seeds. It should be stressed that a node may appear in more than one chain. This explains why six of the nine chains collapse into one main component (see Fig 3) and, in effect, why the total number of nodes embedded in each chain exceeds the number of nodes within the link-tracing network. Further, seed 1 and 9 were unsuccessful in generating additional participants and that seed 4 had a chain length of only three. For comparative purposes, Table 7 displays the demographic composition of these network chains as well as of the link-tracing network. Network chains are assigned their corresponding seed. Several things can be noticed in Table 7. Firstly, approximately 61% of the total link-tracing network nodes (*i*.*e*., 656) and more than a third of the total participants (117 out of 303) are embedded in the chain stemming from seed 2. Secondly, chains vary in composition of the country wherein referrals currently reside. For instance, the chains stemming from the second and sixth seeds are dominated by people with residence in Spain, whereas chains stemming from the third and fifth seeds, by people in Romania. Thirdly, gender variation across chains can be noticed, *e*.*g*., in the second seed chain, the sample has a majority of females (61%), while, in the third seed chain, the sample is about equally split between males and female. Fourthly, across chains and link-tracing network, the average distance of any pair of nodes variates between one and six.

**Table 7:** The demographic composition of the link-tracing network chains.

	Seed#1	Seed#2	Seed#3	Seed#4	Seed#5	Seed#6	Seed#7	Seed#8	Seed#9	link tracing network
*Type of participants*										
Migrants in Spain	1	107	16	3	1	41	9	11	1	147
Non-migrants	0	56	64	0	11	10	1	0	0	138
Return migrants	0	14	3	0	0	0	2	0	0	18
*Type of network members*										
Participants	1 (14%)	177 (27%)	83 (34%)	3 (23%)	12 (34%)	51 (23%)	12 (34%)	11 (37%)	1 (25%)	303 (28%)
Non-participants	6 (86%)	479 (73%)	163 (66%)	10 (77%)	23 (66%)	166 (77%)	23 (66%)	19 (63%)	3 (75%)	765 (72%)
*Type of network members by country*										
*Spain*										
Participants	1 (14%)	107 (16%)	16 (7%)	3 (23%)	1 (3%)	41 (19%)	9 (26%)	11 (37%)	1 (25%)	147 (14%)
Non-participants	3 (43%)	264 (40%)	45 (18%)	9 (69%)	9 (26%)	117 (54%)	14 (40%)	17 (57%)	3 (75%)	382 (36%)
Total network members	4 (57%)	371 (56%)	61 (25%)	12 (92%)	10 (29%)	158 (73%)	23 (66%)	28 (93%)	4 (100%)	529 (50%)
*Romania*										
Participants	0 (0%)	70 (11%)	67 (27%)	0 (0%)	11 (32%)	10 (4%)	3 (9%)	0 (0%)	0 (0%)	153 (14%)
Non-participants	3 (43%)	215 (33%)	118 (48%)	1 (8%)	14 (40%)	49 (23%)	9 (26%)	2 (7%)	0 (0%)	383 (36%)
Total network members	3 (43%)	285 (44%)	185 (75%)	1 (8%)	25 (71%)	59 (27%)	12 (34%)	2 (7%)	0 (0%)	539 (50%)
*Type of network members by sex*										
Males	0 (0%)	257(39%) ^a^	126 (51%) ^a^	2 (15%)	6 (17%)	69 (32%) ^a^	12 (34%) ^a^	10 (33%)	2 (50%) ^a^	436 (41%)
Females	7 (100%)^a^	399 (61%)	120 (49%)	11 (85%) ^a^	29 (83%) ^a^	148 (68%)	23 (66%)	20 (67%) ^a^	2 (50%)	632 (59%)
*Type of network members by country and sex*										
*Spain*										
Male participants	0 (0%)	33 (5%)	1 (0%)	0 (0%)	0 (0%)	12 (6%)	3 (9%)	4 (13%)	1 (25%)	40 (4%)
Male non-participants	0 (0%)	94 (14%)	20 (8%)	2 (15%)	1 (3%)	34 (16%)	4 (11%)	5 (17%)	1 (25%)	133 (12%)
Female participants	1 (14%)	74 (11%)	15 (6%)	3 (23%)	1 (3%)	29 (13%)	6 (17%)	7 (23%)	0 (0%)	107 (10%)
Female non-participants	3 (43%)	170 (26%)	25 (10%)	7 (54%)	8 (23%)	83 (38%)	10 (29%)	12 (40%)	2 (50%)	249 (23%)
*Romania*										
Male participants	0 (0%)	36 (5%)	42 (17%)	0 (0%)	1 (3%)	4 (2%)	0 (0%)	0 (0%)	0 (0%)	83 (8%)
Male non-participants	0 (0%)	94 (14%)	63 (26%)	0 (0%)	4 (11%)	19 (9%)	5 (14%)	1 (3%)	0 (0%)	180 (17%)
Female participants	0 (0%)	34 (5%)	25 (10%)	0 (0%)	10 (29%)	6 (3%)	3 (9%)	0 (0%)	0 (0%)	73 (7%)
Female non-participants	3 (43%)	121 (18%)	55 (22%)	1 (8%)	10 (29%)	30 (14%)	4 (11%)	1 (3%)	0 (0%)	203 (19%)
***Chain volume (# nodes)***^b^	7 (100%)	656 (100%)	246 (100%)	13 (100%)	35 (100%)	217 (100%)	35 (100%)	30 (100%)	4 (100%)	1068 (100%)
***Longest distance from the seed***	1	16	14	3	5	13	6	8	1	-
***Average distance from the seed***	1	7.9	8.6	1.8	2.7	6.3	3.5	3.0	1	-
***Average distance in the network (SD)***	1.0 (0.0)	6.2 (3.5)	5.9 (3.4)	1.7 (0.8)	2.1 (1.0)	5.1 (2.9)	2.6 (1.4)	2.9 (1.9)	1.0 (0.0)	6.1 (3.5)
***Proportion in the link-tracing network*** ^b^	.7%	61.4%	23.0%	1.2%	3.3%	20.3%	3.3%	2.8%	.4%	-

Percentages are computed column-wise based on each chain’s volume (number of nodes). In some cases, these may exceed 100%.

^a^ Within computations, seeds are included in the class of participants.

^b^ Some of the participants appear in more than one seed-chain. For this reason, summation of chain volumes exceeds the total number of network members (1,068).

### The network of networks

**Fig 6:**
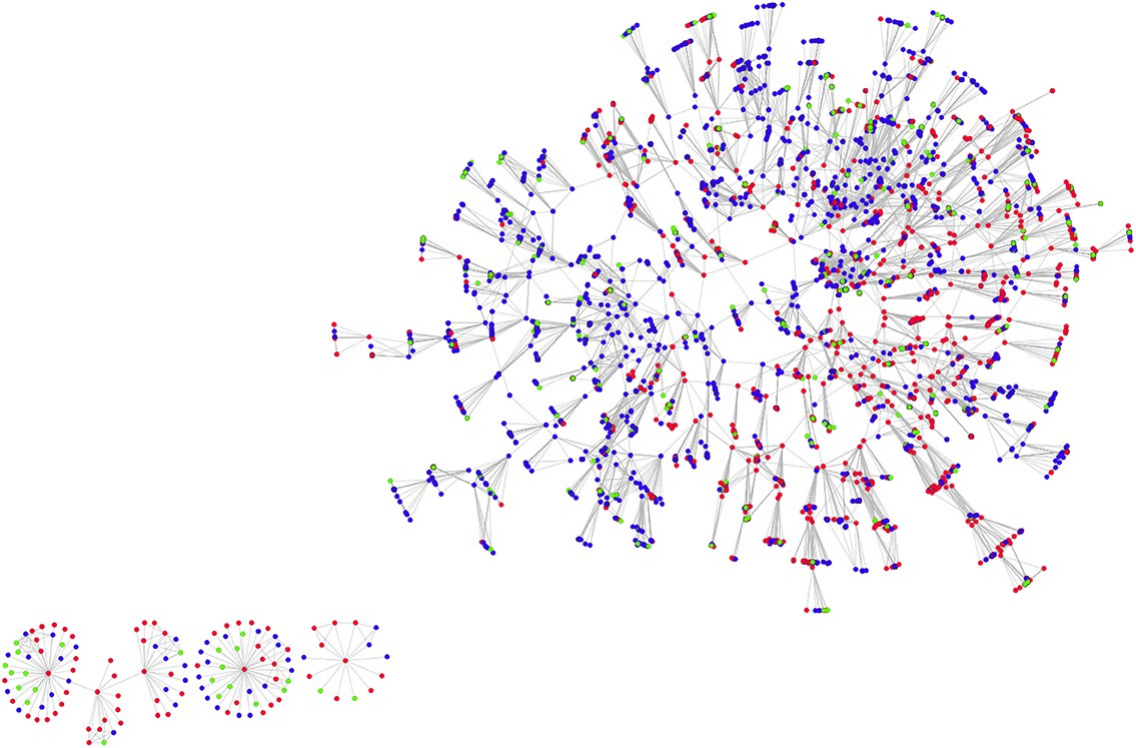
The network of networks. The network has 4,855 nodes, 5,477 arcs and 2,540 undirected ties. Colors indicate network nodes’ place of living, *i*.*e*. red (Castellón, Spain), blue (Dâmbovița, Romania), and green (other countries). The network data was visually encoded with visone (*stress minimization* node layout) [57].

In the remainder of this section, we report results on the compositional and structural features of the *network of networks*, *i*.*e*., the network built by interconnecting the link-tracing network and the personal networks of participants (specifically, their references to personal contacts, alters, other than the referrals). The network of networks consists of 4,855 nodes (participants, referrals, and alters), 5,477 arcs (nomination ties) and 2,540 symmetric ties (ego-perceived alter-alter ties). Fig 6 illustrates the network of networks using a hair-ball layout (*i*.*e*., *stress minimization node-layout* available with visone [57]). Node colors mark the place of residence (*red* for Castellón or other places in Spain, *blue* for Dâmbovița or other places in Romania, and *green* for places in other countries). Despite the large volume of nodes, four components can be identified, *i*.*e*. a main (giant) component that accounts for 95% of all nodes, and three small components stemming from seeds 1, 4 and 9, accounting for the other 5%.

**Fig 7:**
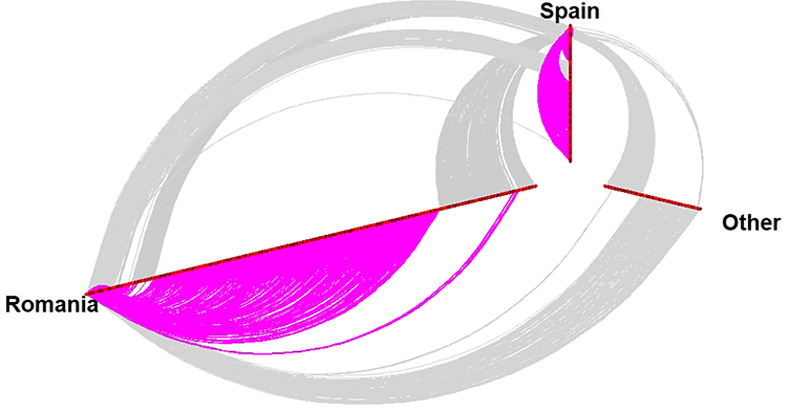
The network of networks (ties split on residence). The hiveplot illustrates how the 4,855 nodes and the 5,477 nomination ties within the network of networks are partitioned on residence (countries wherein people live: Spain, Romania and other countries). Each node is positioned on the axis based on its rank (*out-degree* or the number of people they nominated), under the principle of *first served* (for equal degrees, nodes are placed based on the assigned numbering in the dataset). Magenta ties indicate *within-country* social connections (*i*.*e*. 1,524 ties connect people living in Spain, and 2,237, people living in Romania), whereas gray ties, *between-country* connections (*i*.*e*. 1,133 ties connect Spain and Romania, 223 ties connect Romanians living in Spain to Romanians living in other countries, and 360 ties connect Romanians living in Romania to Romanians living in other countries). The axis illustrating the “other countries” class of nodes lacks within-ties by design. Nodes assigned to this axis were not interviewed, but only nominated by participants (contacts they have in their personal networks, either relatives, friends or acquaintances). The axes are unstandardized to emphasize differences in the volume of nodes.

Fig 7 visually encodes the network of networks using a hive-plot format. Nodes are distributed on three axes based on their residence. Magenta ties indicate edges within the same country, *i*.*e*., Spain–Spain (1,524 ties) or Romania–Romania (2,237). The axis illustrating the “*other* countries” class of nodes lacks within-ties by design. Nodes assigned to this axis were not interviewed, but only nominated by the participants (contacts they have in their personal network, either relatives, friends or acquaintances). The gray colored lines indicate inter-country ties. There are 1,133 ties connecting Spain and Romania. Also, 223 ties are sent from Spain and 360 ties from Romania to Romanians living in other countries, suggesting that interviewees are not only a part of the binational corridor (Spain-Romania) but are also connected to other corridors. Adding the name generator of family, friends and acquaintances in other places is a novelty of our methodology, which could expand our view on transnationalism beyond a single TSF. The axes are unstandardized to emphasize differences in the volumes of nodes. There are 1,656 nodes assigned to the “Spain” axis (1,049 females– 63%), 2,638 nodes to the “Romania” axis (1,337 females– 51%) and 561, to the “Other countries” axis (269 females– 48%). On each axis, node placement was ranked by the out-degree (the number of nominations elicited by each participant), while for equal out-degree, placement was based on the indexation in the database.

To better understand these implications, Table 8 reports the number of *alters* (people embedded in the personal networks of the participants) elicited by the respondents. The respondents elicited on average 16 alters (the maximum by design was 40), summing up to a total of 4,816. Inspecting the average scores across alter categories and respondent classes, several things are noteworthy. Firstly, migrants in Spain, nominated three times more acquaintances and friends living in Castellón or Spain, compared to the return migrants and seven times than non-migrants in Romania. Secondly, people with migration experience in Castellón (either currently living there, or returnees) nominated more relatives living in Spain compared to non-migrants. Thirdly, migrants in Castellón nominated almost half of the relatives and roughly four times less friends living in Dâmbovița or Romania compared to returnees and non-migrants. These three observations were to be expected, as people mostly interact with others who live close to them. Fourthly, overall, migrants in Spain nominated fewer acquaintances and friends and relatives compared to the other two classes of respondents.

**Table 8:** Number of alters nominated within the name generators, split by type of participants.

	Migrants in Spain		Returned migrants		Non migrants		ORBITS study participants	
	(n = 147)		(n = 18)		(n = 138)		(n = 303)	
		Mean (SD)		Mean (SD)		Mean (SD)		Mean (SD)
Acquaintances and friends, living in Castellón or other places in Spain	824	5.6 (2.3)	29	1.6 (2.3)	110	0.8 (1.3)	963	3.2 (3.0)
Acquaintances and friends, living in Dâmbovița or other places in Romania	258	1.8 (2.2)	136	7.6 (3.2)	1138	8.2 (3.3)	1,532	5.1 (4.3)
Acquaintances and friends, living in other countries	113	0.8 (1.2)	17	0.9 (1.8)	152	1.1 (1.5)	282	0.9 (1.4)
*Acquaintances and friends*	1,195	8.1 (4.7)	182	10.1 (3.8)	1,400	10.1 (4.1)	2,777	9.2 (4.5)
Family, living in Castellón or other places in Spain	348	2.4 (1.6)	38	2.1 (1.9)	161	1.2 (1.7)	547	1.8 (1.7)
Family, living in Dâmbovița or other places in Romania	387	2.6 (1.9)	100	5.6 (2.5)	719	5.2 (2.1)	1,206	4.0 (2.4)
Family, living in other countries	103	0.7 (1.1)	11	0.6 (1.3)	172	1.2 (1.6)	286	0.9 (1.4)
*Family*	838	5.7 (3.4)	149	8.3 (2.9)	1,052	7.6 (3.1)	2,039	6.7 (3.4)
***Elicited alters***	**2,033**	**13.8 (7.2)**	**331**	**18.4 (3.9)**	**2,452**	**17.8 (5.9)**	**4,816**	**15.9 (6.8)**

The observation that is, however, of most interest in terms of the novelty of the current methodology is that people with no migration experience in Spain (non-migrants) nominated as least as many people living in other countries compared to people living in Spain. Had we not added this name generator, we would have underestimated non-migrants’ transnationality. Notably, we would have known that 12,9% of all nominations of non-migrants were to people living in Spain (excluding the people living in other countries from the equation), but we would not have known that 24,3% of all their nominations lived in other countries. This is, however, an important outcome, as it implies that non-migrants are influenced by multiple TSFs at the same time, which can influence their identities, subsistence strategies, migration intentions and other variables. Moreover, non-migrants nominated more relatives (almost double) and slightly more friends living in other countries compared to the participants with Spanish migration experience.

Table 9 presents a general overview on the structural features exhibited by the *network of networks* as well as by the *link-tracing network* and *chain-networks*. These features are displayed on three levels of measurements: basic elements, dyadic and network level. The network of networks has 4,855 nodes, 5,477 directed ties (referrals or nominations) and 162 mutual dyads, *i*.*e*., referrals who nominated their referees in their personal networks or back as referrals (this number of mutual dyads is almost insignificant given that the total number of possible reciprocated dyads in the network is of more than 11 million; we excluded from computations the 2,540 undirected ties representing the alter-alter ties). The extremely low dyadic reciprocity can also be noticed in the other layers (the link-tracing and chain networks, where it may be more expected) of the network of networks.

**Table 9:** Structural characteristics for chain networks, link-tracing network and the network of networks.

	Seed 1^a^	Seed 2	Seed 3	Seed 4^a^	Seed 5	Seed 6	Seed 7	Seed 8	Seed 9^a^	Linktracing	Linktracing^b^	Network of networks^d^
***Basic elements***												
Nodes	7	656	246	13	35	217	35	30	4	1,068	303	4,855
Ties	6	732	269	12	34	240	36	36	3	1,187	382	5,477
***Dyads***												
Mutual dyads	0	17	2	0	0	5	2	4	0	24	24	162
Asymmetric dyads	6	698	265	12	34	230	32	28	3	1,139	334	5,153
***Network level measurements***^d^												
Network density	0.143	0.002	0.004	0.077	0.029	0.005	0.030	0.041	0.250	0.001	0.004	0.000
Indegree centralization	2.8%	0.4%	0.8%	0.7%	0.1%	1.3%	2.9%	6.4%	11.1%	0.3%	0.9%	0.2%
Outdegree centralization	100.0%	2.1%	3.2%	36.8%	18.3%	5.1%	18.1%	20.7%	100.0%	1.3%	1.6%	0.9%
Degree centralization	50.0%	1.1%	1.6%	15.5%	9.4%	2.5%	9.3%	10.3%	50.0%	0.7%	0.7%	0.5%
Betweenness centralization	0.0%	0.5%	1.1%	5.6%	2.3%	2.4%	4.1%	4.0%	0.0%	0.2%	0.6%	0.3%
Share of the main component	100%	100%	100%	100%	100%	100%	100%	100%	100%	96%	98%	95%

^a^ Measurements for these networks do not have a substantial meaning due to their small number of nodes. However, we did the computations for illustrative purposes. Readers should address the corresponding measurements with caution.

^b^ A variant of the link-tracing network wherein only the participants (the respondents) were kept.

^c^ The network level measurements should be interpreted in association with the number of basic elements in each network.

^d^ In the *Network of networks*, we filtered out the 2,540 symmetric ties (alter-alter ties).

Concerning the *network density* (observed by expected ties), the ratio decreases as the number of nodes increases (large networks are generally scarce in terms of ties). Moreover, as reported throughout the paper, all the ERGM models indicated that the number of observed ties is lower compared to the one expected by chance alone (*see* Tables 6 and 8). Looking at the results reported in the network level measurements section of the Table 9, we notice that, generally, the networks exhibit low levels of centralization. In a nutshell, network centralization would be high when nodes vary severely in degree distributions (some nodes would have significantly more ties compared to others—the “all roads lead to Rome” effect). It is noteworthy that we detected statistically significant (*p* < 0.001) popularity effects in the ERGM models applied to the link-tracing network of participants. However, that does not conflict with the overall centrality measurements computed in Table 9. Further, across the valid networks: a) *indegree centralization* (centralization that takes into account only the number of times a node gets nominated) varies between 2.0% and 11.1%; b) *outdegree centralization* (centralization that accounts only for the nominations made by a node) varies between 0.9% and 20.7%; c) *degree centralization* (centralization that take into account the number of times a node nominates and gets nominated by other nodes) varies between 0.5% and 10.3%; d) betweenness centralization (centralization that accounts for the number of times a node is placed, in the network, between other two nodes) varies between 0.2% and 4.1%.

The last network-level measurement reported in Table 9 concerns the number of components (in a component, all pairs of nodes are reachable). Nodes that compose the seed (chain) networks are by design part of the same component. The *network of networks* has four components (the main component includes 95% of the 4,855 nodes). The link-tracing networks also have four components: *the link-tracing network of participants* (wherein non-participants were filtered out) has a main component that includes 98% of the 303 nodes, whereas the *full link-tracing network* has a main component that includes 96% of the 1,068 nodes.

## Discussion and conclusions

We employed an innovative bi-national community link-tracing methodology to sample from a transnational social field, encompassing Romanians living in a migration destination place (Castellón, Spain), as well as their connections living in a Romanian migration sending community (Dâmbovița). Our paper aimed to describe the implementation of this methodology as well as to evaluate the structural and compositional features of the resulting sample (link-tracing networks), to guide future research. We start this section by briefly summarizing the main findings. In our study, roughly, one out of three contacted people accepted to participate. The number of people invited to the study was similar in both sites. The socio-demographic profile of the participants was overall similar irrespective of the destination or the origin. On average, respondents were in their early forties, had a high-school diploma, were married with children, held a job and shared the Orthodox religion. Generally speaking, the resulting sample comprised on average economically active people.

Regarding the properties of the resulted link-tracing network of participants, we found that 2-path and triple closure effects are statistically significant, indicating the tendency to nominate close contacts. This result is in line with the NSIT study, wherein triad closure was reported as a significant predictor of connection between nodes [12]. Also, we discovered that nominations are patterned by homophily in sex (women tended to nominate women, and men tended to nominate men) and residence (the participants tended to nominate more participants living in their country of residence). Such assortative mixing is, nevertheless, generally expected in the organization of social networks [19]. For example, high levels of gender homophily were observed in the NSIT study too [12]. In other research, the social ties among Chinese migrants in Tanzania were patterned by province of origin and ownership sector of employment [11].

We further found that referrals were generally reluctant to participate and, in effect, the link-tracing advanced with difficulty. For instance, only one of the nine seeds was dominant at the level of the entire network (*i*.*e*., a third of participants and two-thirds of the elicited names were embedded in only one seed-chain). Moreover, three seed-chains out of nine displayed, on average, more than six waves. Also, the overall behavior of the participants during the study was rather similar. Most respondents recruited fewer participants than expected, and were nominated by (very likely) close referrals. A similar situation is reported in the NSIT study, wherein the ERGM models support statistically significant negative estimates for sociality [12].

Referring to the low response rates exhibited in our study, we would comment that these are typical for surveys (including here the ones based on face-to-face questionnaire administration). While peer recruitment may generally lead to higher rates of responses, the relatively high social and institutional distrust observed in Romania [58] may have recruitment factors low. Higher incentives might have increased survey response, but in turn may have caused ethical concerns.

Analyzing the composition of the personal networks of the participants, we derive several important sample features. First, Romanian migrants in Spain report in their networks three times more acquaintances and friends living in Castellón or other places in Spain, than elsewhere. The prevalence of these weak ties suggests that the social organization of their life in Spain may be characterized by social incorporation [59]. Moreover, their families are living transnational lives or are geographically split between origin and destination. For example, respondents living in Castellón have half of their families at the destination and half in Dâmbovița. In the case of the returned migrants, evidence suggests that these people remain an active part of the TSF by connections to relatives, friends and acquiantances still living in Castellón. Supplementary, the sample data indicate that all participants report social contacts living in other countries (other than Romania and Spain). This showcases that they are simultaneously part of other migration corridors. Put it differently, we may claim that the Castellón–Dâmbovița migration corridor is embedded in a wider global network, wherein it is connected to other bi-national corridors.

Our study falls within the emerging efforts of applying social network research to the investigation of international migration [60–66]. Sampling from TSFs is inherently difficult, rigorous, complex and entails vast resources. Not surprisingly, similar studies are scant, despite their surmised valuable contribution to advancing knowledge on human mobility flows. Despite of the network character of migration, social network analysis methods have been rarely appointed and put to work to understand community structure [10]. Therefore, little is known yet about the structure and the composition of the transnational networks. In this line, our endeavor displays a methodological innovation. Our dataset and methodology allow not only the study of TSFs but also the composition of the personal networks embedded in these fields. For instance, we can compare the composition of migrants’ personal networks to those of returned migrants and non-migrants. Consequently, our research framework methodologically mixes personal network design (the study of an individual and her surrounding pattern of social contacts) with socio-centric network design (the study of the whole network embedding a population of interest). This is a substantial advancement compared to similar network or attribute data studies. Particularly, previous network oriented methodologies either have focused on socio-centric networks intended to capture trans-border connectivity, or have been limited to re-creating the social universe around migrants and their contacts [5].

Moreover, conventional studies (surveys, interviews, etc.), given their attribute oriented nature [67], cannot reconstruct the social structures supporting the migration routes. Traditional survey or in-depth interview questionnaires lack the ability of thoroughly tracking the social contacts of migrants and their embeddedness in structures along with other migrant peers, returned migrants and non-migrants. Conversely, our methodological framework quantitatively describes and explores the role of networks in the migration processes at an individual, micro-level. Network data collection techniques are suitable for scrutinizing the composition and structure of migrants’ personal networks. Uniquely, it permits the study of transnational networks, such as the measurement of structural assimilation and transnationalism, of the network determinants of cultural and economic integration, of social support, and the circulation of remittances and reversed remittance circulation. Therefore, our study has numerous implications. For instance, our data may be useful in testing and developing theories referring to transnationalism (how migrants build social fields that cross geographic, cultural and political borders, how remittances and reversed remittances circulate, how mobility networks influence the social support available to migrants), social assimilation and integration [64], or how communication is being managed within TSFs [25]. At the same time, it may play a sheer role in assessing the impact of the persistence of transnational ties upon integration at the destination [25]. The implications of our findings are wide and diverse. Building the migration networks may provide insights about how migration is patterned and produced [66]. Examining the structures underlying the migration corridors is essential for the development of national and supra-national public policies [68] and for enhancing the coordination of transnational policies. Given that international migration affects social cohesion in destination countries, our data are extremely useful for understanding the processes of integration / assimilation in the new societies of residence (how migrants integrate at destination) [61]. The identification of the structural properties of the migration networks (e.g., the density of the personal networks, the role of the brokers etc.) is a valuable input for understanding how migrants manage their support networks, increase their social capital or control information flows.

The practical challenges of implementing binational link-tracing designs imply both technical (study-related) and contextual (external to the research) aspects. The selection procedures and the complexity of the data collection instruments assume a certain level of understanding (or education) and cooperation from the targeted respondents as well as their access to communicational technologies (mobile or smart phones for eliciting alters and nominations). Multi-sited network research, by its nature, is conducted in different cultural and social contexts and under distinct institutional arrangements. Respondents’ behavior and social realities radically change from one site to the other. Consequently, different field-work strategies are required from the researchers, while operating under the same methodology. All these challenges are expected to impact upon the participation rate and the quality of the collected data. Also, the application of link-tracing sampling may be improved in various ways. One of these ways refers to the data anonymization process that currently imposes tremendous efforts. Based on our fieldwork experience, we consider that link-tracing sampling from migrant populations can be improved. To our knowledge, currently available software packages are not customized for the process of generating and controlling acronyms used for data anonymization. Human creation and manipulation of acronyms is prone to errors which, by consequence, it increases the necessary time for data cleaning and processing, and threatens the accuracy of datasets. This aspect is of critical importance especially for the creation of the transnational networks by inter-connecting the personal networks of the study participants. Due to the high specificity of sampling from TSFs, existent software package tools, such as *RecordLinkage* in R, have only a limited efficacy in assessing and solving for conflicting acronyms (*e*.*g*., cases wherein one individual has different acronyms, or several individuals have the same acronym).

TSFs are instrumental in explaining socio-cultural phenomena (e.g., the direction in which remittances circulate, ethnic identifications) and in providing systematic representations of human mobility (i.e., a piece of evidence not available in the information about migration flows that it is periodically posted by international and national agencies). Link-tracing sampling makes visible the hidden social structures connecting transnational places and provides access to unique information about migration networks. Due to its complexity and intricacies, empirical applications of link-tracing sampling from TSFs are still coalescing. In effect, further research work is needed to reach a comprehensive understanding about how to better manage the challenges of modeling TSFs. In this context, we are aware that our study may display at least two limitations. First, notably, the resulting sample comprised, on average, economically active citizens. It remains for future work to ascertain whether these people are representative for the TSF or an artefact of the methodology. Second, it is not evident to what degree our sample allows generalization. Clarification of these aspects may be achieved by replicating the current methodology to a different but culturally similar bi-national migration corridor. In this regard, future work is already underway as we currently replicate the methodology to a different immigrant sending community in Romania (Bistrița-Năsăud) and destination area in Spain (Roquetas de Mar). This replication is meant to bring forth better practical mechanisms for controlling homophily in nomination patterns and to increase the knowledge about the factors impeding the outreach of seed chains. Other possible avenues for future research refer to assessing the impact that institutions and organizations have on supporting specific TSFs (the multi-level organization of transnational networks) [35]. Further, the data collection process could be extended from a bi-national link-tracing sampling approach to a multi-national frame-work (conducting interviews in more than two countries). Last, collecting longitudinal data would increase the understanding of how *multi-level multinational networks* develop over time (multi-layered networks embedding different social entities–individuals and organizations, identified in multiple countries and connected by transnational relationships).

In conclusion, in this paper, we described the implementation and the results of an innovative bi-national link-tracing methodology [12, 13, 25] designated to sample from TSFs. We believe that our work will be useful for researchers interested in examining the role of networks in migration processes, and that our analyses of the resulting networks inspire others to control the resulting data in detail. Hopefully, the methodology and data contribute to a better understanding of human mobility trajectories. From another angle, the methodology may even be applicable to other social fields that, while not two-sited, may include two or more actor types (*i*.*e*., the social field of science dissemination, which includes scientists and social agents). Consequently, it may be of interest to a wider community of scholars even beyond migration studies.
